# Lower Respiratory Tract Infection in a Renal Transplant Recipient: Do not Forget Metapneumovirus

**DOI:** 10.1155/2012/353871

**Published:** 2012-11-14

**Authors:** N. Noel, B. Rammaert, J. Zuber, N. Sayre, M. F. Mamzer-Bruneel, M. Leruez-Ville, L. Mascard, M. Lecuit, O. Lortholary

**Affiliations:** ^1^Université Paris Descartes, Sorbonne Paris Cité and Hôpital Necker-Enfants Malades, Service des Maladies Infectieuses et Tropicales, Centre d'Infectiologie Necker-Pasteur, Institut Imagine, AP-HP, 149 rue de Sèvres, 75743 Paris Cedex 15, France; ^2^Université Paris Descartes, Sorbonne Paris Cité and Hôpital Necker-Enfants Malades, Service de Transplantation Rénale, AP-HP, 75743 Paris Cedex 15, France; ^3^Université Paris Descartes, Sorbonne Paris Cité and Hôpital Necker-Enfants Malades, Laboratoire de Microbiologie, Unité de Virologie, AP-HP, 75743 Paris Cedex 15, France; ^4^Groupe Microorganismes et Barrières de L'hôte, Institut Pasteur, Inserm Avenir U604, Paris, France

## Abstract

Human metapneumovirus (h*MPV*) is emerging as a cause of a severe respiratory tract infection in immunocompromised patients. h*MPV* pneumonia has only been seldom reported in nonpulmonary solid organ transplanted patients, such as renal transplant recipients. We report here a case of a 39-year-old patient presenting with fever, cough, and interstitial opacities on CT scan diagnosed as a nonsevere h*MPV* pneumonia 11 years after a renal transplantation. Infection resolved spontaneously. Differential diagnosis with *Pneumocystis* pneumonia was discussed. We review the medical literature and discuss clinical presentation and detection methods that can be proposed in solid organ transplant recipients.

## 1. Introduction

Respiratory viruses such as *respiratory syncytial virus* (*RSV*), *influenza*, *parainfluenza* viruses (*PIV*), and *adenovirus* are commonly associated with mild to severe symptoms, depending on the immune status. Human Metapneumovirus (h*MPV*) was the sixth most frequent viral infection in patients hospitalized for respiratory illness [1]. h*MPV* is a nonsegmented, enveloped, negative single-stranded RNA virus [2] responsible for lower respiratory tract infections (LRI), especially in extreme ages [3, 4]. It has a seasonal distribution and occurs mainly in winter and spring, with an incubation period usually between 4 and 6 days [5]. h*MPV* is now widely recognized as an opportunistic infection in immunocompromised hosts such as hematopoietic stem cell transplant (HSCT) and pulmonary transplant recipients, leading to a significant respiratory morbidity [6]. Although its detection is not yet routinely performed, h*MPV* appears to account for 9% of acute pneumonia in patients with haematologic malignancies (including HSCT), in a similar proportion to *RSV* [7]. This rate is close to that reported in lung transplant recipients, ranging from 6% to 12% of LRI [6, 8].

In contrast, it has been seldom reported in other SOT settings such as renal transplantation [9].

## 2. Case Report

A 39-year-old patient with an 11-year history of kidney transplantation for severe amyloidosis was referred to the Centre d'Infectiologie Necker Pasteur for acute fever for 2 days. After 8 years of transplantation, he was treated for graft rejection by corticosteroids. Clinical course was uneventful, except for recurrent prostatitis. His current immunosuppressive regimen consisted of mycophenolate mofetil (250 mg b.i.d) and ciclosporin (50 mg b.i.d). Basal creatinine serum level was 130 *μ*mol/L. On admission, temperature was 39°C, and clinical examination was unremarkable except for sore throat and rhinorrhea. Biological analyses showed an elevated C-reactive protein blood level (157 mg/L, normal <5 mg/L) but normal blood leukocytes and neutrophil counts. Blood lymphocyte count was low (0.94 G/L), with CD4+ T cells accounting for 39.5% of total lymphocytes (0.372 G/L). HIV serology was negative. Blood and urine cultures were sterile and initial chest radiograph was normal.

**Figure 1:**
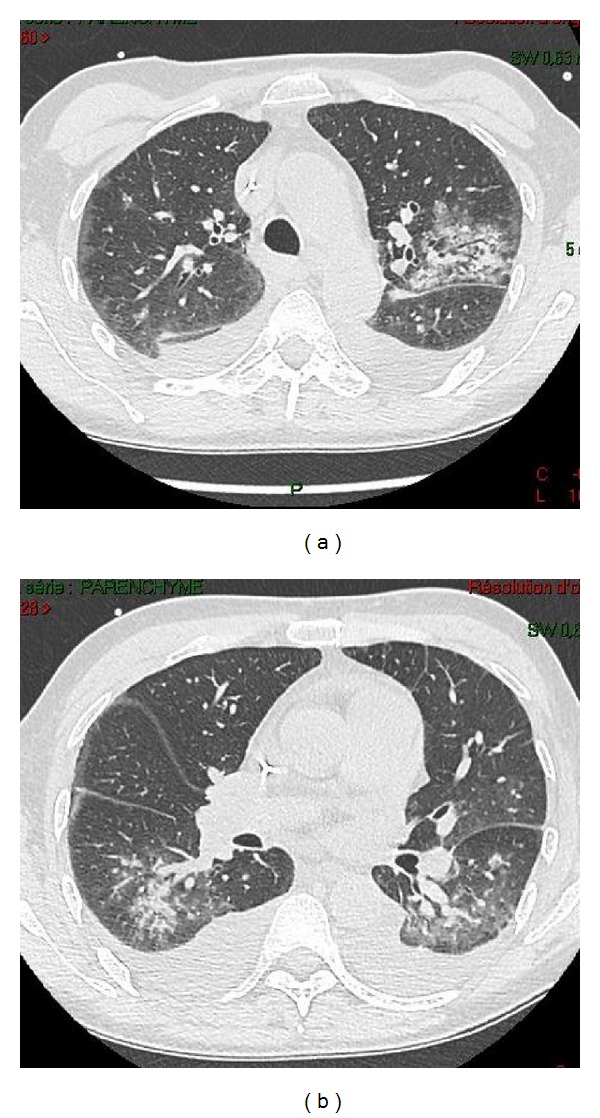
Thoracic computed tomography scan of the patient, showing bilateral and diffuse extension of the infection. (a) Bilateral subpleural and peribronchial infiltrates with bilateral pleural effusion in the superior lobes. (b) Disseminated alveolointerstitial infiltrates in the basal areas.

Nonproductive cough without dyspnea or chest pain appeared on day 3 of hospitalization. Oxygen saturation in ambient air was 92%. Chest auscultation was normal. As the cough increased, a thoracic computed tomography (CT) scan was performed on day 6 and revealed bilateral ground glass infiltrates mainly located in subpleural and peripheral areas, associated with bilateral pleural effusion (Figure 1). No mediastinal adenopathy was seen. Because of clinical and radiological presentation suggesting *Pneumocystis jirovecii* pneumonia, trimethoprim-sulfamethoxazole was initiated the same day.

Nasopharyngeal aspirates were screened by direct immunofluorescence with specific monoclonal antibodies to *RSV*, *influenza virus A* and *B*, *PIV*, *adenovirus,* and h*MPV* (Argène, Verniolle, France) on day 7. Immunofluorescence was strongly positive for h*MPV* and negative for other viruses. Blood cultures and *S. pneumoniae* and *L. pneumophila* urinary antigen detections were negative. As *Pneumocystis *pneumonia was initially suspected, bronchoalveolar lavage (BAL) fluid analysis performed on day 11 demonstrated 450.10^3^ cells/mL (macrophages 72%, neutrophils 17%, and lymphocytes 11%). Microbiological studies did not reveal any bacterial or fungal microorganisms. Gomori-Grocott staining for *Pneumocystis jiroveci *detection, indirect immunofluorescence, and polymerase chain reaction (PCR) for *P. jiroveci* were negative. h*MPV* was also detected in BAL fluid by direct immunofluorescence. As all microbiological investigations were negative except for h*MPV*, antibiotics were discontinued; respiratory symptoms spontaneously improved within 6 days. Thus, decreased immunosuppression or other medications such as ribavirin or intravenous immunoglobulin were not considered. The patient was discharged on day 14.

## 3. Discussion

This is the second h*MPV* pneumonia in a kidney-transplanted recipient described in the literature. The first reported case was a severe LRI requiring transient intensive care unit stay [9]. It occurred three years after kidney transplantation, while receiving immunosuppressive regimen consisting of ciclosporine (125 mg b.i.d), azathioprine (75 mg/d), and prednisone (10 mg/d). Compared to this case, our patient had mild symptoms, mainly cough and upper respiratory symptoms. He was also less immunosuppressed without corticosteroids regimen.

In solid organ transplanted patients, h*MPV* is responsible for LRI and may lead to hospitalization and significant respiratory illness in up to 63% of cases [6, 8]. As initial clinical symptoms are nonspecific, thoracic CT scan can be more helpful than chest X-ray, which is less sensitive. Consolidation, nodular infiltrates, and pleural effusions may be seen. Subpleural and basal areas are usually observed, and bilateral locations are seen in 50% of cases, as in our case [10]. Whereas crazy paving, network of a smooth linear pattern superimposed on an area of ground-glass opacity, is unusual, bronchiectasis is common, up to 68% in the series by Wong et al. [10]. 

Of note, lymphopenia, as noticed in our patient, is the most common feature reported in HSCT patients with h*MPV*, accounting for 73% of patients in one series [7]. This illustrates that although innate immune responses are stimulated upon h*MPV* exposure, adaptive immunity also appears important to control h*MPV*. As for other paramyxoviruses, the matrix proteins are involved in the induction of proinflammatory and Th1 responses by dendritic cells and macrophages (i.e., production of interleukin-2 and interferon-*γ*) [11]. Inflammation may cause diffuse alveolar damage and hyaline membrane formation as shown by histopathology investigations [12].

Apart from other respiratory viral infections occurring in SOT recipients, differential diagnoses of h*MPV*-associated LRI include severe bacterial and fungal pneumonitis, particularly *Pneumocystis* pneumonia. Ribavirin, previously shown active in a mouse model of infection [13], has been suggested as a potential antiviral therapy in HSCT and lung transplant recipients with hMPV-associated LRI [14, 15]. In our case and in the other case of the literature [9], ribavirin was not used because the diagnosis was made retrospectively after the patient's spontaneous clinical improvement.

In conclusion, h*MPV* has to be considered as a potential cause of LRI in kidney transplant recipients and may mimic *Pneumocystis* pneumonia. A prompt recognition would have avoided antibiotic use and further diagnostic studies such as bronchoscopy. Its early detection using immunofluorescence and/or RT-PCR must be proposed routinely in transplantation settings. In addition, early recognition could improve the implementation of appropriate infection control practices to prevent viral spread of this potential life-threatening infection in immunocompromised patients.
